# Clinical Course of Patients Treated for Advanced Ovarian Carcinoma without Surgical Intervention

**DOI:** 10.1371/journal.pone.0055645

**Published:** 2013-01-30

**Authors:** Ram Eitan, Haim Krissi, Hanoch Levavi, Gad Sabah, Yoav Peled

**Affiliations:** Gynecologic Oncology Division, The Helen Schneider Hospital for Women, Rabin Medical Center, Petach Tikva, affiliated with Sackler Faculty of Medicine, Tel Aviv University, Tel Aviv, Israel; Baylor College of Medicine, United States of America

## Abstract

**Objective:**

To describe the clinical course and outcome of patients with non-surgically-treated advanced ovarian cancer attending a single institute.

**Methodology/Principal Findings:**

We reviewed the medical charts of all patients with advanced epithelial ovarian cancer who underwent chemotherapy at a tertiary medical center between January 2005 and December 2010 but were never operated. Data on patient characteristics, disease course, and outcome were collected from patient files. Sixteen patients met the inclusion criteria. Eight (50%) were diagnosed with apparent FIGO stage IIIC disease, and 8 with stage IV. Five patients (31%) achieved a complete clinical response, and 11 (69%) achieved a partial response. Among the complete responders, the median disease-free interval was 8 months (range 7–11 months). In all of them, the disease recurred and second-line chemotherapy was administered. Of them, four (80%) achieved a second complete response. Partial responders had up to four lines of chemotherapy, with continued disease progression. The median overall survival of the whole group was 19.5 months, and of the complete responders, 28 months.

**Conclusions/Significance:**

Most patients with advanced ovarian carcinoma who will not undergo surgery respond only partially to first-line chemotherapy. Having no surgery is associated with a short disease-free interval.

## Introduction

Surgery is central in the treatment of ovarian carcinoma. Surgical staging procedures are employed for apparent stage 1 disease, and extensive cytoreductive surgery is believed to be vital for long-term survival in patients with advanced epithelial ovarian cancer [1], [2]. Patients left with minimal residual disease fare better than patients who remain with bulkier lesions [3]–[5]. Whether the maximal surgical effort should be made upfront, at diagnosis, or after neo-adjuvant chemotherapy is still unclear [6]. Over the last two decades, several studies have addressed the use of chemotherapy prior to surgery in women with advanced epithelial ovarian cancer [7]–[9], and recently, a large randomized trial suggested that neoadjuvant chemotherapy is not detrimental to survival relative to upfront surgery [10].

The finding that some patients with advanced peritoneal disease remain with only minimal or even no residual disease by interval cytoreduction raises the question of the added value of surgery to the clinical course in these cases. This issue is important, because not all patients with advanced ovarian cancer are eligible for surgery owing to background medical conditions that rule out laparotomy and extensive resection or poor performance status, and others refuse surgery for personal reasons. Our search of the literature yielded no contemporary studies of patients with ovarian carcinoma treated without surgery at all. Thus, the aim of the present study was to describe the clinical course and outcome of patients with advanced epithelial ovarian carcinoma attending a single institute who did not undergo surgery after responding to first-line upfront chemotherapy.

## Materials and Methods

We reviewed the medical charts of all women attending a tertiary university-affiliated medical center between January 2004 and December 2010 for the treatment of newly diagnosed advanced ovarian carcinoma. Those patients who received chemotherapy with no subsequent attempt at cytoreduction were included in the study. Data on background and demographics, pathologic diagnosis, initial outcome and follow-up, treatment history, response, and survival were collected from the charts. Response was assessed by CA125 levels and computed tomography (CT). Complete response was diagnosed if patients had a normalized CA125 level and no pathologic space occupying lesion on CT. All scans were compared to initial scans before treatment.

### Statistical analysis

Data analysis was performed with the SPSS v15.0 software. Standard two-sided statistical tests were used to identify the clinical characteristics that significantly predicted survival.

## Results

During the study period 265 patients with ovarian carcinoma were treated at our institution. Sixteen patients aged 41 to 82 years who did not receive chemotherapy were enrolled. Their clinical characteristics are shown in Table 1. In 15 patients, the diagnosis of ovarian carcinoma was based on cytologic findings of malignant cells in ascites or pleural effusion samples, followed by cell block with immunohistochemistry to establish the mullerian origin of the tumors and in one patient by biopsy study of a sample from the omentum. All patients had invasive epithelial ovarian carcinoma and none had borderline malignancy. Eight patients (50%) were diagnosed with apparent International Federation of Gynecology and Obstetrics (IFGO) stage IIIC disease, and 8, with stage IV disease. All 16 patients were started on first-line chemotherapy. In 10 patients with extensive disease, surgery was planned after neo-adjuvant chemotherapy. Surgery was not performed in 5 cases due to patient refusal. Five additional patients had a deterioration of background conditions, worsening their performance status. Surgery was contraindicated in these patients. The other six patients did not undergo primary surgery because of low performance status at diagnosis (Table 1).

**Table 1:** Patient characteristics at diagnosis of ovarian carcinoma.

Apparent FIGO stage	No. (%)
IIIC	8 (50%)
IV	8 (50%)
Diagnosis by	
Cytology	15 (94%)
Histology	1 (6%)
Reason for neoadjuvant	
Extent of disease	10 (62.5%)
Performance status	6 (37.5%)
Karnofsky performance status at diagnosis	
80–100	2 (12.5%)
50–70	10 (62.5%)
40	4 (25%)
Initial chemotherapy regimen	
Carboplatin only	6 (37.5%)
Carboplatin and paclitaxel	10 (62.5%)

FIGO-International Federation of Gynecology and Obstetrics.

**Figure 1:**
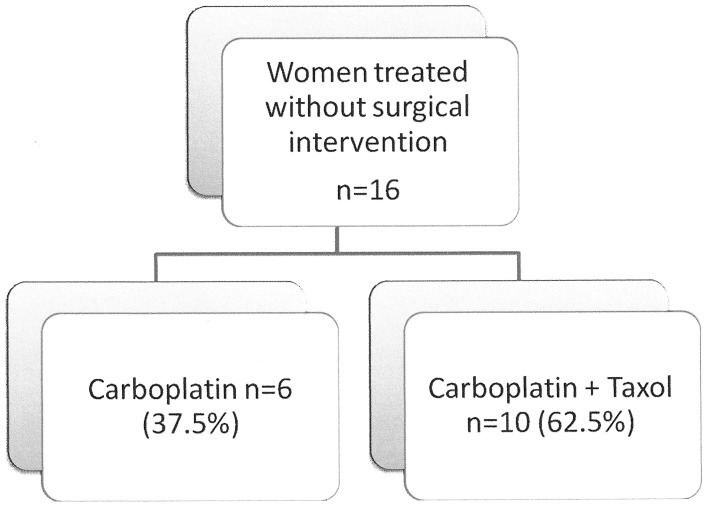
First line chemotherapy for patients never treated surgically.

First-line chemotherapy is described in Figure 1. Seven patients (43%) received 6 courses of chemotherapy, and 3 patients each received 7, 8, and 10 courses. Single agent carboplatin was administered at the discretion of the treating physician as per patient overall condition and preference.

**Figure 2:**
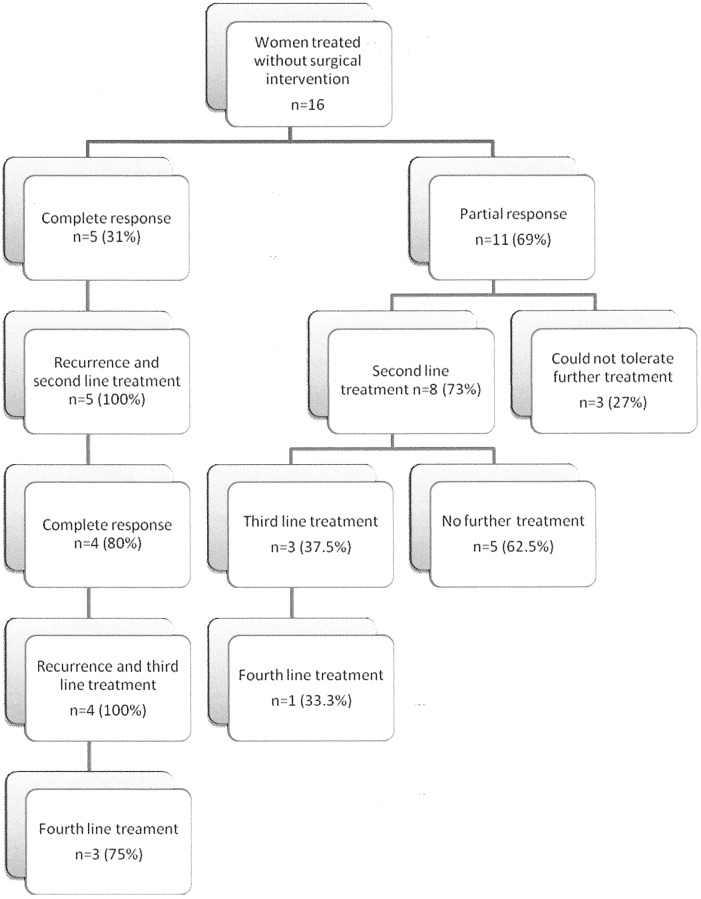
Patients with ovarian carcinoma treated without surgical intervention – flowchart of expected outcomes.

CA125 levels normalized by the third cycle of chemotherapy in 6 patients (37%) and by the sixth cycle in 10 patients (62%). Five patients (31%) achieved a complete clinical response, and 11 patients (69%) achieved a partial clinical response (Figure 2). All patients with an elevated CA125 after cycle 6 had measurable disease on CT scan or physical examination.

For the 5 complete responders, the median disease-free interval (DFI) was 8 months (range 7–11 months). The disease recurred in all 5 and was treated by second-line chemotherapy. Four patients (80%) achieved a second complete response. Of the 11 partial responders to first-line chemotherapy, 8 (73%) received second-line chemotherapy, with eventual progression of the disease. All 8 patients also received third-line treatment and 3 received fourth-line treatment. The disease continued to progress during third- and fourth-line treatment.

As per the division protocol, patients failing the combination of carboplatin and paclitaxel receive single agent liposomal doxorubicin, followed by topotecan, gemcitabine and docetaxel. Patients recurring within 6 months receive the same protocols. Patients recurring beyond 6 months are first re-challenged with single agent carboplatin and then treated as per the same protocol.

Follow-up ranged from 4 to 48 months. As of last day of follow up, 9 patients (56%) had died of the disease and 7 (44%) were alive with disease. The median overall survival (OS) for the whole patient group was 19.5 months, and for the complete responders, 28 months.

## Discussion

This study evaluated the outcome of patients with advanced epithelial ovarian cancer treated with chemotherapy only, without cytoreductive surgery. Using chemotherapy as the only modality of first line therapy, only a minority of patients, 30%, achieves complete remission. Those patients, who do achieve a complete response, had a DFI of 8 months. Previous studies reported a similar DFI for patients who achieved a second remission after second-line chemotherapy without secondary cytoreduction [11]. Standard care, including surgery followed by adjuvant chemotherapy in newly diagnosed patients with advanced disease has been shown to give patients 20 to 24 month DFI and 40 to 65 month overall survival [12], [13]. These groups of patients are not comparable, of course, but the difference is notable. Some of these patients were eligible for surgery but refused and we show that the omission of surgery might be detrimental to survival in these patients. We don’t believe the use of single agent carboplatin is the cause of lower overall survival because 2 of the 5 patients who achieved complete response received single agent therapy.

A unique group of patients are those having a complete clinical response after initial first line chemotherapy. We found that the DFI and OS of these patients are comparable to the values reported in the neoadjuvant EORTC/NCIC trial [10]. In this study, patients with advanced ovarian disease were randomized to either upfront chemotherapy or cytoreduction. DFI was 12 months in both groups, and OS was 29 and 30 months respectively. In the present study, separate analysis of the 5 patients with a complete response to first-line chemotherapy yielded an OS of 28 months. We still do not know how to correctly identify these patients. It would be interesting to see a subgroup analysis of the patients in the EORTC/NCIC trial looking at the patients achieving a complete pathological response during surgery. Also, we urge our colleagues to report larger retrospective analyses of patients in the same clinical situation.

Unlike other peritoneal malignancies, such as pancreatic carcinoma and gastric cancer, which are treated only with chemotherapy when diagnosed at an advanced stage and are associated with poor prognosis because of low chemo-sensitivity [14], epithelial ovarian cancer is typically chemo-sensitive and most patients treated with the combination of surgery and chemotherapy achieve a complete response. Patients treated only with first-line chemotherapy do not achieve complete response at the same rate. Some researchers suggested that ovarian carcinoma is not a uniform disease and requires individually tailored therapy but unfortunately, at this time, there is no way to distinguish between these patient groups.

Our study is limited by its retrospective design and small sample size. Common practice guidelines cannot be drawn from these small numbers and caution should be used extrapolating to the individual patient. Nevertheless, this is a unique patient population that has not been assessed in the recent literature in terms of disease course. Our results demonstrated that patients with advanced epithelial ovarian cancer who cannot or will not undergo surgery have a lower probability of reaching complete remission and a shorter DFI than patients treated with chemotherapy and surgery. Those who achieve complete remission have a similar OS to surgically treated patients after neo-adjuvant chemotherapy, but they are the minority. Most patients will require multiple lines of sequential chemotherapy, with no DFI.

We believe that our findings are unique and important as they establish the baseline for survival and response for patients with advanced ovarian carcinoma without surgical intervention.
